# Treatment guided by rapid diagnostic tests for malaria in Tanzanian children: safety and alternative bacterial diagnoses

**DOI:** 10.1186/1475-2875-10-290

**Published:** 2011-10-06

**Authors:** George Mtove, Ilse CE Hendriksen, Ben Amos, Hedwiga Mrema, Victor Mandia, Alphaxard Manjurano, Florida Muro, Alma Sykes, Helena Hildenwall, Christopher JM Whitty, Hugh Reyburn

**Affiliations:** 1 National Institute for Medical Research, Amani Centre, Muheza, Tanga, Tanzania; 2 Mahidol-Oxford Research Unit, Mahidol University, Bangkok 10500, Thailand; 3 Teule Hospital, Muheza, Tanzania; 4 Kilimanjaro Christian Medical Centre, Moshi, Tanzania; 5 Division of Global Health, Karolinska Institute, Nobels V13, 17177 Stockholm, Sweden; 6 London School of Hygiene and Tropical Medicine, Keppel St, London WCIE7HT, UK; 7 Joint Malaria Programme, PO. Box 2228, KCMC, Moshi, Tanzania

## Abstract

**Background:**

WHO guidelines for the treatment of young children with suspected malaria have recently changed from presumptive treatment to anti-malarial treatment guided by a blood slide or malaria rapid diagnostic test (RDT). However, there is limited evidence of the safety of this policy in routine outpatient settings in Africa.

**Methods:**

Children 3-59 months of age with a non-severe febrile illness and no obvious cause were enrolled over a period of one year in a malaria endemic area of Tanzania. Treatment was determined by the results of a clinical examination and RDT result, and blood culture and serum lactate were also collected. RDT-negative children were followed up over 14 days.

**Results:**

Over the course of one year, 965 children were enrolled; 158 (16.4%) were RDT-positive and treated with artemether-lumefantrine and 807 (83.4%) were RDT-negative and treated with non-anti-malarial medicines. Compared with RDT-positives, RDT-negative children were on average younger with a lower axillary temperature and more likely to have a history of cough or difficulty in breathing. Six (0.6%) children became RDT-positive after enrolment, all of whom were PCR-negative for *Plasmodium falciparum *DNA at enrolment. In addition, 12 (1.2%) children were admitted to hospital, one with possible malaria, none of whom died. A bacterial pathogen was identified in 9/965 (0.9%) children, eight of whom were RDT-negative and one was RDT-positive, but slide-negative. Excluding three children with *Salmonella typhi*, all of the children with bacteraemia were ≤12 months of age. Compared to double-read research slide results RDTs had a sensitivity of 97.8% (95%CI 96.9-98.7) and specificity of 96.3% (95%CI 96.3-98.4).

**Conclusions:**

Use of RDTs to direct the use of anti-malarial drugs in young children did not result in any missed diagnoses of malaria although new infections soon after a consultation with a negative RDT result may undermine confidence in results. Invasive bacterial disease is uncommon in children with non-severe illness and most cases occurred in infants with a current fever.

## Background

The features of malaria overlap with those of other common illnesses and, where diagnosis is based on clinical history and examination alone, this often results in a large degree of overtreatment for malaria [1]. For many years this has been considered as a price worth paying to ensure prompt treatment of malaria in young children, but over the last decade a number of important trends have driven a movement towards parasitological testing to guide the use of anti-malarial drugs [2]. These include the replacement of older anti-malarial drugs with the more expensive artemisinin combination therapy (ACT), the need to minimize selection pressure for drug-resistant strains of *Plasmodium falciparum*, the need for more reliable surveillance data as malaria has declined in many areas of Africa, and the relative neglect of alternative diagnoses to malaria [3-7].

In parallel to this, accurate and affordable rapid diagnostic tests for malaria (RDTs) have become available over the last decade [8] and the 2010 WHO malaria treatment guidelines now propose that wherever possible all patients suspected of malaria should be tested by blood slide or RDT and only those with a positive test result should receive anti-malarial treatment [9]. While this should lead to a number of benefits, there is still relatively little direct evidence that non-malarial treatment in young febrile children in highly or moderately endemic areas is safe, with a concern that a significant number of malaria cases may be missed. The risks of 'test negative malaria' are consistently raised by clinicians cautious about this approach [10]. In addition clinicians need more evidence on the probabilities of different non-malarial diagnoses, especially those that require treatment with antimicrobial agents.

This study was conducted in a malaria endemic area of Tanzania to document the clinical outcomes and blood culture results of children with febrile illness whose treatment had been guided by the results of a commonly used RDT.

## Methods

### Study area

The study was conducted in the mother and child health (MCH) clinic of Teule Hospital, the District Designated Hospital of Muheza District, Tanzania. This serves a predominantly rural population exposed to perennial transmission of malaria with seasonal peaks coinciding with the short and long rainy seasons. In common with much of East Africa, malaria incidence has declined in recent years; in1990 parasite prevalence in children under the age of five years was approximately 80% and this had declined to 30% in 2008 [11,12]. In order to facilitate patient follow-up the study area was defined as residence in one of the 85 villages from which there had been > 10 paediatric admissions to Teule Hospital in 2006.

### Clinical and laboratory procedures

All children presenting to the MCH clinic were screened for the following inclusion criteria; age 3-59 months and resident within the study area, reported fever in the previous two days or current axillary temperature ≥37.5 C, no obvious non-malarial cause of fever and no known chronic illness. Children with evidence of severe illness were admitted to the paediatric ward and excluded from the study.

Following consenting procedures, a medical history and clinical examination were undertaken according to IMCI guidelines. Venous blood was drawn for culture (2-5ml), haemoglobin concentration (Hemocue™, Angelholm, Sweden), serum lactate (Lactate-Pro™, Arkray Inc, Kyoto, Japan), HRP-2 based RDT for *P. falciparum *(Paracheck™, Orchid Biomedical, Mumbai, India).

Giemsa-stained blood slides were prepared from venous blood and independently double-read, with discordant results (either positive/negative discordance or > 2-fold density difference above 400 parasites/μL) resolved by an independent third reading. Microscopists were blinded to RDT results. Parasite densities were calculated from the geometric mean of the two closest counts of asexual parasites/200 white blood cells assuming 8,000 WBC/μl. Blood for culture was inoculated into a BactALERT™ Paediatric-fan bottle (bioMérieux, France) and incubated in the BacT/ALERT 3D automated microbial detection system. Cultures that flagged positive were identified by standard methods. Cultures positive for non-cryptococcal yeasts, coagulase-negative Staphylococcus, Micrococcus, Corynebacterium, Bacillus sp. or viridans group Streptococci were considered contaminants and classified as 'negative culture' unless a pathogenic organism was also isolated. The laboratory participated in a bacteriology external quality assurance programme coordinated by a reference laboratory in Moshi, Tanzania that participates successfully in external quality assurance programmes of the College of American Pathologists. At all visits, a malaria RDT, blood slide and blood spot for parasite DNA identification by polymerase chain reaction (PCR) were obtained according to established methodology [13].

Children were treated according to IMCI guidelines but anti-malarials or other medication with anti-malarial activity was avoided in RDT-negative children. RDT-negative children were seen and checked on Days 2, 7 and 14 following the initial visit (Day 0) with home visits the following day (and up to 2 more days until seen) if they did not attend. RDT-positive children were treated with artemether-lumefantrine (ALu) as recommended in the Tanzanian Guidelines for Malaria Diagnosis and Treatment (2006). RDT-positive children were not routinely followed up but results of positive blood cultures were promptly reviewed by a study clinician and, if the isolate was judged to be a pathogen, home visits were conducted for three consecutive days or until the child was seen and appropriate treatment and follow up was arranged. Children were also seen at additional times on parental demand and admissions to the hospital ward for children in the study were identified and clinical details obtained from hospital records.

### Data management and analysis

Data were double-entered in MS Access and analysed in Stata-11. Simple proportions and confidence intervals were calculated for prevalence of pathogens, and risk of malaria in RDT-negative cases at follow-up. Sensitivity and specificity of Paracheck against the gold standard of double-read research miscroscopy was undertaken on baseline samples. Groups were compared using Wilcoxon rank-sum test for continuous variables and chi-square test for proportions. The study was able to detect prevalence of pathogens in 800 RDT-negative patients at 1% with 95%CI 0.5-2% and a 5% prevalence with a 95%CI 3.6-6.6%.

### Ethics

Written informed consent was obtained from the parent or caretaker of each child, or witnessed verbal consent if the parent could not read. The study was approved by the Ethical Review Boards of the National Institute for Medical Research in Tanzania (NIMR/HQ/R.8a/Vol.IX/785) and the London School of Hygiene & Tropical Medicine in the UK (LSHTM ethics # 5523).

## Results

**Figure 1:**
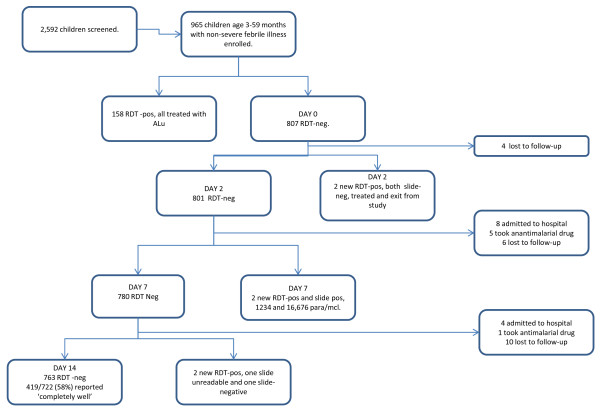
**Children in the study by RDT result**.

The study was conducted over one year from April 2009; 2592 children were screened, and 965 were eligible and enrolled (Figure 1). Of these, 158 were RDT-positive and treated with ALu and 807 were RDT-negative and received non-anti-malarial treatment. Baseline characteristics by RDT result are shown in Table 1; children who were RDT-negative were on average younger with a lower axillary temperature and more likely to have a history of cough or difficulty in breathing (Table 1).

**Table 1:** Clinical and laboratory characteristics of RDT positive compared to RDT-negative children at enrolment into the study

	RDT-Positive, n = 158	RDT-Negative, n = 807	p*
Age in months, median(mean)	26 (28)	16 (20)	< 0.001
Days ill, median(mean)	3 (3.8)	3 (3.3)	0.002
Haemoglobin g/dl, median(mean)	9.7 (9.6)	10.5 (10.4)	< 0.001
Resp. rate/min, median (mean)	48 (46.3)	48 (47.4)	0.16
WHO criteria of pneumonia, n (%)^†^	48 (30.4%)	417 (51.6%)	< 0.001
Diarrhoea or vomiting, n (%)	40 (25.3%)	155 (19.2%)	0.079
Axillary temperature, °C, median(mean)	38.1 (38.1)	37.4 (37.5)	< 0.001
Blood lactate, mmol/L, median(mean)	2.2 (2.5)	1.8 (2.0)	< 0.001

*Wilcoxon rank-sum test for continuous variables or chi-square test for proportions

^† ^Cough or difficulty in breathing plus raised respiratory rate for age (> 40 breaths/min 12-59 months, > 50 breaths per minute 2-11 months of age)

RDTs had a sensitivity of 98.6% (95%CI 97.8-99.3) and specificity of 97.3% (95%CI 96.3-98.4) when compared to double-read research slide results; 14.3% of blood slides were positive with a negative predictive value of RDT results of 99.8% (95%CI 99.4-100).

Of the 807 patients who were RDT-negative, 765 (94.8%) remained in the study for the 14 days of follow-up at which time 461 (60.3%) of whom were reported by the caretaker to be 'completely well'. Of 42 patients who exited before the end of the study, the parents of 6 children administered an anti-malarial drug during the 14-day follow-up period and all of these were RDT-negative at their next research visit and a further six children became RDT-positive after enrolment and were treated with ALu, two of whom were slide-positive with 16,676 and 1,234 asexual parasites/μl respectively and all of whom were PCR-negative for *P. falciparum *DNA at enrolment. Twenty children were lost to follow-up of whom 6 (30%) were untraceable and 14 (70%) had left the study area but were reported (by telephone or friends) to be well at the time they exited the study. In addition, 12 children were admitted to hospital for whom details are provided in Table 2.

**Table 2:** Diagnosis, treatment and outcome of 12 children admitted to hospital during the 14 days of follow-up.

Age (months)	Diagnosis or clinical syndrome	RDT result	Blood slide result	Treatment	Outcome
**24 m**	Acute diarrhoea	Negative	Negative	Quinine and gentamycin	Discharged well
**7 m**	Acute diarrhoea	Negative	Negative	IV rehydration	Discharged well
**22 m**	Acute diarrhoea	Negative	Negative	IV rehydration	Discharged well
**8 m**	Suspected pneumonia	Negative	Negative	Cotrimoxazole	Discharged well
12 m*	Clinical diagnosis of malaria	Negative	Not available	Quinine	Discharged well
**8 m**	Acute diarrhoea	Negative	Not available	IV rehydration	Discharged well
**7 m**	Febrile convulsion	Not done	Not available	Paracetamol	Discharged well
24 m*	Fever and vomiting	Not done	Not available	Quinine	Discharged well
18 m*	Fever and vomiting	Not done	Not available	Quinine	Discharged well
**29 m**	Fever, convulsion, prostration	Positive	Not available	Quinine	Discharged well
18 m*	Suspected dysentery	Negative	Negative	IV rehydration	Discharged well
**18 m**	Pneumonia	Not done	Negative	Amoxycillin	Discharged well

*Admitted to non-study facility

Diagnoses made by the study clinician at the first consultation are shown in Table 3. Almost half (383, 47.5%) of RDT-negative and 29 (18.4%) of RDT-positive children met WHO criteria for non-severe pneumonia and were prescribed amoxicillin and an additional 34 (4.2%) of RDT-negative and 19(12.0%) of RDT-positive children also met these criteria but were not treated with amoxicillin. There was no evidence of a trend of the proportion of children with raised respiratory rate or a recent history of 'cough or difficulty in breathing' with month of the study that might indicate increased prescriber confidence to withhold antibiotic treatment (p = 0.37 and p = 0.43 respectively). While 73% of respiratory rate values were even, there was no apparent preference for values at or above cut-off levels; the commonest values were 36/min (9.7% of values) and 48/min (15.4% of values).

**Table 3:** Diagnoses made by examining clinician at enrolment by RDT result

	RDT-Negative			RDT-Positive		
	**First diagnosis**	**Secondary diagnoses**	**Total (%)**	**First diagnosis**	**Secondary diagnoses**	**Total (%)**
Upper resp. tract infection	150	5	155 (14.2)	4	6	10 (3.6)
Non-specific fever	182	6	188 (17.2)	0	0	0
Diarrhoea	72	40	112 (10.2)	2	7	9 (3.2)
Pneumonia	375	8	383 (35.0)	20	9	29 (10.6)
Malaria	0	0		127	31	158 (57.0)
Anaemia	0	206	206 (18.8)	0	59	59 (21.3)
Bronchitis/wheeze	0	3	3 (0.3)	0	0	0
Other	28	19	47 (4.4)	5	7	11 (4.1)
TOTAL	807	287	1,094 (100)	158	119	277 (100)

A bacterial pathogen was identified from blood culture in 9/965 (0.9%) children in the study, 8 of whom were RDT-negative and one was RDT-positive but slide-negative (Table 4). In addition, an organism judged to be a contaminant was isolated in 56 (5.8%) of cultures. All of the bacteraemic children were successfully treated as outpatients and the majority (7/9) were treated with amoxicillin at the first consultation before the blood culture result was available. Clinical and laboratory features associated with bacteraemia are shown in Table 5, none of which reached 5% significance although 'current fever' (measured axillary temperature > = 37.5C) was positively associated with bacteraemia (p = 0.08) and with sensitivity and specificity of 77.8% (95%CI 75.2-80.4) and 46.5% (95%CI 43.4-49.6) respectively. In addition, with the exception of children with *S. typhi*, all of the children with bacteraemia were 12 months of age or younger

**Table 4:** Clinical and laboratory data on the 9 children whose blood grew a bacterial pathogen.

Age (mon)	Blood slide/RDT	Organism	Summary clinical and laboratory data	*Antibiotic susceptible	Antibiotic resistant
8	Neg/Neg	*Acinitobacter Iwoffli*	7 days cough, temp 36.8°, pulse 138/min, resp. rate 63/min, lactate 1.4 mmol/L	amox, cip, ceft, gen, nal	chlor, cip
8	Neg/Neg	β-Haemolytic *streptococcus*	3 days cough, temp 37.2°pulse 144/min, resp. rate 56/min, lactate 1.1 mmol/L	eryth, ceft.	Pen, amox.
7	Neg/Neg	Gram-negative coccus	3 days cough, temp 37.4°, pulse 167/min, resp. rate 64/min, lactate 1.8 mmol/L	No data	
8	Neg/Neg	*Haemphilus influenzae*	3 days cough and coryza, temp 37.7°, pulse 150/min, lactate 3.1 mmol/L	amox, ceft, gen	chlor, cip, nal
10	Neg/Neg	Non-Typhi *Salmonella*	2 days cough, temp 39.5°, pulse 156, resp rate 64, lactate 2.0 mmol/L	amox, chlor, cip, ceft, gen, nal	
43	Neg/Neg	*Salmonella Typhi *	2 days cough, temp 39.2, pulse 145/min, resp rate 39/min, lactate 1.9 mmol/L	cip, ceft, gen	chlor, cotrim, amox
38	Neg/Neg	*Salmonella Typhi *	4 days diarrhoea, temp 38.1°, pulse 120/min, resp rate 42/min, lactate 3.3 mmol/L	chl, cip, cef, gen	amox, cotrim.
47	Neg/Neg	*Salmonella Typhi *	7 days febrile illness, temp 38.8, pulse 140/min, resp rate 36/min, lacate 0.9 mmol/L	chl, cip, cef, nal	cotrim, amox
12	Neg/Pos	*Staphylococcus aureus*	1 day cough, temp 37.4°, pulse 163/min, resp. rate 52/min, lactate 0.9 mmol/L	chl, eryth, gen	cotrim, amox

*Anti-microbial agents tested for in vitro susceptibility: amoxicillin (amox), chloramphenicol (chlor), cotrimoxazole (cotrim), ciprofloxacin (cip), erythromycin (eryth), oxacillin(pen), ceftriaxone (ceft), nalidixic acid (nal).

**Table 5:** Clinical and laboratory features of children by presence of a bacterial pathogen isolated from blood culture.

	Bacterial pathogen, n = 9	No bacterial pathogen, n = 956	**p**^†^
Median (mean) age in months	10 (20.0)	17 (21.0)	0.56
Days of illness, median (mean)	3 (3.6)	3 (3.4)	0.68
Cough or difficulty in breathing* (%)	7 (77.8%)	601 (62.8%)	0.35
Diarrhoea or vomiting* (%)	1 (11.1%)	194 (20.3%)	0.49
Median (mean) axillary temp,°C	38.1 (38.2)	37.5 (37.6)	0.08
Respiratory rate, median (mean)/min	52 (51.8)	48 (47.2)	0.15
Median (mean) blood lactate mmol/L	1.9 (2.1)	1.8 (2.0)	0.94
Median (mean) aemoglobin, g/dL	10.2 (10.4)	10.4 (10.3)	0.93
WHO criteria of non-severe pneumonia (%)	5 (55.6%)	460 (48.1%)	0.65

* Symptoms reported at any time in the previous 2 days

## Discussion

While age, haemoglobin level and presence of respiratory symptoms provide some guide to diagnostic probability, clinical predictors of malaria are still insufficient to guide treatment. The advent of RDTs provides the potential to target anti-malarial treatment on a scale that has hitherto been impossible. However, doubts remain in the minds of many clinicians about the safety of not treating parasite-negative patients for malaria, and these have probably contributed to the observation from a number of studies in Africa that up to half of those with a negative rapid test or blood slide result for malaria are treated with anti-malarials anyway. Additionally the question 'if it is not malaria what is it?' is important for framing a rational diagnostic and treatment strategy. Appropriate use of anti-microbial drugs is a key issue given the high mortality of children admitted to hospital with invasive bacterial disease but whose treatment is restricted by high levels of resistance to commonly used agents [14].

This study adds to other evidence that anti-malarial treatment based on parasitological testing misses few true cases of malaria. Njama-Meyer *et al *used expert microscopy to guide the treatment of 2,359 illness episodes in children under the age of 10 years in a malaria-endemic area of Uganda; fewer than 1% of initial blood slide readings were false-negatives, and only 13 (0.8%) of 1,602 slide-negative illness episodes became positive over the following even days, and none of these was severely ill [15]. D'Acremont *et al *followed up 603 RDT-negative children who had not been treated for malaria and only three children developed a positive result in the following week, all of whom were successfully treated [16].

Although the study results are reassuring, the policy of RDT-directed treatment for suspected malaria represents a substantial change in the delivery of routine care in Africa and some questions still remain. Firstly, it is impossible to distinguish between 'new' or 'missed' diagnoses of malaria during routine follow-up of RDT-negative patients. As observed in the current study, a significant minority of children will be admitted for malaria within days of being seen with a non-severe febrile illness and a negative RDT result. Although the PCR primers used in this study were not the most sensitive, the results suggest that the malaria diagnoses following enrolment into the study were 'new' infections and that it is rational and safe to treat RDT-negative children for a non-malarial illness. However, the perception of clinicians and parents may be that a child with malaria has been denied the best treatment as a result of application of new guidelines with RDTs. Given evidence of a strong preference for a diagnosis of malaria among parents and clinicians [17], such an interpretation is quite likely and may undermine efforts to implement the policy of RDT-directed anti-malarial treatment. To guard against this, clinicians should be reminded that 'no test is perfect' and to advise parents to bring their child back if they remain unwell. Secondly, there is still a possibility that presumptive treatment for malaria in all children with fever might provide a benefit due to intermittent prophylaxis that might protect against malaria and/or anaemia. Recent results of trials of intermittent treatment for malaria in children suggest that this is possible, and a cluster randomized trial of presumptive compared to RDT-directed anti-malarial treatment is in progress [18].

The commonest indication for anti-microbial treatment in our study was the IMCI diagnosis of non-severe pneumonia. While the overlap between malaria and pneumonia has been previously described both in severe and non-severe illness, the proportion of children meeting WHO criteria for non-severe pneumonia was higher than expected from previous studies [15,19-21]. Preferential recording by study clinicians in order to justify prescribing an anti-microbial drug seems unlikely as the number of children meeting the WHO definition was slightly higher than the number actually diagnosed. In addition, there was no evidence of a change in the proportion diagnosed with pneumonia as the study progressed, nor was there evidence of clustering of respiratory counts near the cut-off values that define raised levels. It's possible that 'cough or difficulty breathing' was over-interpreted or there may be local variations in the perception or reporting of respiratory problems in children [22]. In addition, the finger-prick undertaken approximately 15 minutes before examination may have resulted in transiently increased respiratory rates.

As new guidelines and RDTs are introduced across Africa it is possible that there will be a compensatory over-use of the IMCI diagnosis of non-severe pneumonia but this will be difficult to assess since there is no gold standard definition of the diagnosis of pneumonia. Even of the true pneumonias some will be viral, including the vaccine-preventable RSV pneumonias, where antibiotics will make little positive impact on outcome [23,24]. In addition, studies of the quality of care suggest that IMCI procedures to diagnose pneumonia, in particular examining the chest and recording respiratory rate, are not well undertaken outside research settings [25,26].

Consistent with at least one other study, we found that bacteraemia was uncommon in children with non-severe illness, especially in children who were RDT-positive [27]. However, blood culture lacks sensitivity and early recognition and treatment of blood stream infections has the potential to avert progression to severe disease with high associated mortality [14,28]. While RDTs can improve targeting of anti-malarial drugs there is no comparable test for bacterial disease. The POC measure of blood lactate was increased in children who were RDT-positive compared to RDT-negative, but was unhelpful in distinguishing between children with and without invasive bacterial infection. While the small numbers of children with bacteraemia in our study reduced the likelihood of finding a statistically significant association with clinical and laboratory features, our findings are consistent with other studies that bacteraemia occurs predominantly in young children, up to three-quarters of whom present with a 'current fever'. However, these indicators lack both sensitivity and specificity and there is still a need for more accurate measures of bacterial infection; devices measuring acute phase proteins such as C-reactive protein or procalcitonin merit further research [29].

This study has a number of inevitable limitations. The study was conducted in hospital outpatients where the patient population may differ in a number of ways from those attending primary health facilities generally. The accuracy of RDT interpretation may have been higher than under routine practice, and certainly higher than routine microscopy in some clinics in the region. Clinicians were aware that they were being observed (in the sense that their findings and prescriptions were being recorded) and may have been more likely to follow IMCI guidelines and to prescribe antibiotics, thus over-estimating antibiotic usage that would occur under similar conditions in routine practice. The clinical care provided in the study followed IMCI guidelines and did not have access to an otoscope or urine examination resulting in possible neglect of otitis media or urinary tract infections. The lack of validating investigations for the IMCI diagnosis of pneumonia has already been discussed.

In conclusion, this study demonstrates that use of RDT's to direct the use of anti-malarial drugs in young children is a safe strategy and did not result in any missed diagnoses of malaria. However, new episodes of malaria do occur within a short period of an initial consultation in malaria endemic areas and these may undermine confidence in parasitological testing for malaria. Invasive bacterial disease is relatively uncommon in children with non-severe illness; while most cases occurred in infants with a current fever there is a need for more specific diagnostic tests to detect these infections at the point of care.

## Conflict of interest

The authors declare that they have no competing interests.

## Authors' contributions

GM was responsible for clinical data collection and drafting the MS, IH was responsible for drafting study documents and supervising data collection, BA was responsible for the laboratory work and provided critical review of the MS, HM was responsible for data collection, VM was responsible for clinical care, AM was responsible for the PCR analysis, FM assisted in the analysis and provided critical review of the MS, AS contributed to the design of the study and provided critical review of the MS, HH assisted in the analysis and writing and provided critical review of the MS, CW initiated the idea for the study and contributed to the writing of the MS, HR was involved in the design of the study, analysed the data and co-wrote the MS. All authors read and approved the final manuscript.
